# Supplementary figures and images for: Deep Learning of Phase-Contrast Images of Cancer Stem Cells Using a Selected Dataset of High Accuracy Value Using Conditional Generative Adversarial Networks
Source: Int J Mol Sci. 2023 Mar 10;24(6):5323. doi: 10.3390/ijms24065323 (PMC10049268; doi:10.3390/ijms24065323)

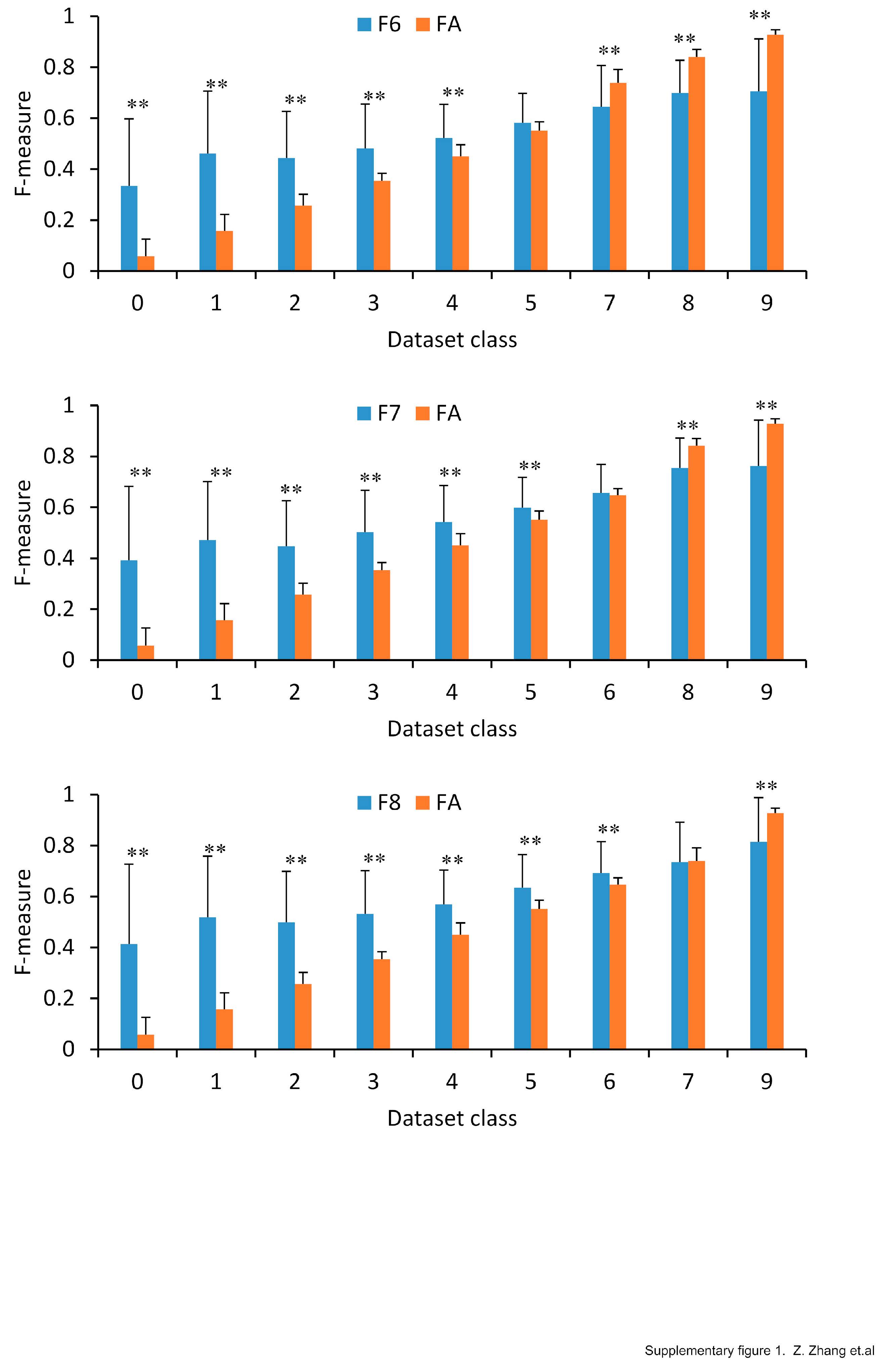

Supplement: Supplementary file 1 [file ijms-24-05323-s001.zip › Figure S1.jpg]
